# Spatiotemporal Patterns in a General Networked Hindmarsh-Rose Model

**DOI:** 10.3389/fphys.2022.936982

**Published:** 2022-06-28

**Authors:** Qianqian Zheng, Jianwei Shen, Rui Zhang, Linan Guan, Yong Xu

**Affiliations:** ^1^ School of Science, Xuchang University, Xuchang, China; ^2^ School of Mathematics and Statistics, North China University of Water Resources and Electric Power, Zhengzhou, China; ^3^ School of Mathematics, Northwest University, Xi’an, China; ^4^ School of Mathematics and Statistics, Northwestern Polytechnical University, Xi’an, China

**Keywords:** HR model, pattern formation, network, matrix, turing instability, delay

## Abstract

Neuron modelling helps to understand the brain behavior through the interaction between neurons, but its mechanism remains unclear. In this paper, the spatiotemporal patterns is investigated in a general networked Hindmarsh-Rose (HR) model. The stability of the network-organized system without delay is analyzed to show the effect of the network on Turing instability through the Hurwitz criterion, and the conditions of Turing instability are obtained. Once the analysis of the zero-delayed system is completed, the critical value of the delay is derived to illustrate the profound impact of the given network on the collected behaviors. It is found that the difference between the collected current and the outgoing current plays a crucial role in neuronal activity, which can be used to explain the generation mechanism of the short-term memory. Finally, the numerical simulation is presented to verify the proposed theoretical results.

## Introduction

Neuron modelling plays a vital role in understanding the brain behavior with its dynamic mechanisms Sun et al. (2018); Wang et al. (2021a),Wang et al. (2021b), Wang et al. (2022); Perc (2006). The HR model is the extended Fitzhugh–Nagumo model, which could exhibit various firing activities (periodic, chaotic, bursting firings, etc.) of the membrane potential Hindmarsh and Rose (1982), Hindmarsh and Rose (1984); Gu and Pan (2015). The dynamic bifurcation of the HR model was calculated and analyzed to illustrate the effects of different parameters on the dynamic behavior Wu et al. (2016). And the HR model with electromagnetic induction by analyzing the stability of equilibrium points was studied to show that electromagnetic induction could regularize chaotic regimes by the number of spikes Goulefack et al. (2022). The HR model with the slow intracellular exchange of calcium ions was investigated to present the effects of the coupling strength and the forcing current on the network behavior Rajagopal et al. (2019). Then the role of delay in the bifurcation behavior was considered in the fractional-order HR model with delay, and the condition for the existence of Hopf bifurcation was present Shi et al. (2020). Also, noise-induced resonances were obtained to describe the response of the HR model to noisy signals and intrinsic oscillations Baltanas and Casado (2002). A networked-organized HR model with delay was developed to explain the spatial relations between neurons and model the process of neural migration Lepek and Fronczak (2018). Although the phenomenon of spatiotemporal patterns was often considered to show the firing activities in a general networked HR model with the delay Umesh and Ambika (2021); Shi et al. (2022); Santos et al. (2017), the interaction mechanism of network nodes remains unclear.

Turing instability is a kind of collective spatial behavior, and the pattern formation could describe the interaction between species in a reaction-diffusion system and explain the biological mechanism (the formation of zebra and zebrafish skin, the nonlocal synaptic interactions) Turing (1990); Kolodina et al. (2021); Kondo et al. (2021). A nonlocal evolution equation was developed to explain color patterns on a guppy fish skin through the bifurcation theory Cygan (2021). The effect of landscape heterogeneity on pattern formation is studied to find the mechanisms of diffusion-driven instabilities in the predator-prey interactions Zaker et al. (2021). And the firing behavior of neurons can be represented by the pattern formation in the network-organized FitzHugh-Nagumo model Zheng and Shen (2020). The elimination of spiral waves is investigated to prevent some brain disorders by the transition between pattern formations in an HR model Eteme et al. (2019). Pattern formation is a well-studied phenomenon in neural field models, which describes the collected groups of neurons and presents the generation of spatial distribution in a dynamical system Kriener et al. (2013). Sometimes, the collective behavior is easier to keep the network in activity Erichsen and Brunnet (2009). Also, short-term memory is a kind of the collective behavior of neurons Zheng et al. (2020). Meanwhile, the network topology is predominant in the critical features of Turing systems Diego et al. (2018). Therefore, the general networked HR model should be considered to show the dynamical mechanism of neuronal activity.

It is well known that the neurons link each other through a network, and the brain activity is the collective behavior of the neurons rather than a single neuron. Pattern formation and bifurcation Tian et al. (2021); Yang (2022); Ma et al. (2021) is a crucial tool to elaborate on the dynamic and biological mechanism of the collective behavior of the neurons. In this paper, an HR model with a random network is considered to show the spatiotemporal patterns of collective behaviors. The effect of the network on pattern formation is presented through the Hurwitz criterion, and the conditions of Turing instability are derived. Then, Hopf bifurcation illustrates the profound impact of network and delay on the collected behavior. It is found that the difference in network topology plays a vital role in neuronal activity. Finally, the numerical simulation explains the generation mechanism of the short-term memory.

## Model Description

In this paper, we consider the following HR model on networks,

$$\begin{array}{c} \frac{dx_{i}}{dt}=y_{i}-ax_{i}^{3}+bx_{i}^{2}+I-z_{i}\left(t-\tau \right)+d_{1}\overset{n}{\underset{j=1}{\sum }}A_{ij}\left(t\right)f\left(x_{j},x_{i}\right),\\ \frac{dy_{i}}{dt}=c-dx_{i}^{2}-y_{i}+d_{2}\overset{n}{\underset{j=1}{\sum }}A_{ij}\left(t\right)g\left(y_{j},y_{i}\right),\\ \frac{dz_{i}}{dt}=r\left(s\left(x_{i}-x_{r}\right)-z_{i}\right), \end{array}$$ (1)

where *x*_*i*_, *y*_*i*_ and *z*_*i*_ (*i* = 1, … , *n*) denote the membrane potential, recovery variable and bursting variable at node (neuron) *i*, respectively. *I* is the external stimulus, *A* is the adjacent matrix of the interaction between nodes, *f* (*x*_*j*_, *x*_*i*_), *g* (*y*_*j*_, *y*_*i*_) is the diffusive function. The equilibrium point of system (1) can be derived from the following Equation 2

$$\begin{array}{c} y_{0}-ax_{0}^{3}+bx_{0}^{2}+I-z_{0}=0,\\ c-dx_{0}^{2}-y_{0}=0,\\ r\left(s\left(x_{0}-x_{r}\right)-z_{0}\right)=0. \end{array}$$ (2)

The linear term of system (1) at (*x*_0_, *y*_0_, *z*_0_) can be expressed as

$$\begin{array}{c} \frac{dx_{i}}{dt}=y_{i}-3ax_{0}^{2}x_{i}+2bx_{0}x_{i}-z_{i}\left(t-\tau \right)+d_{1}\overset{n}{\underset{j=1}{\sum }}A_{ij}\left(x_{j}-qx_{i}\right),\\ \frac{dy_{i}}{dt}=-2dx_{0}x_{i}-y_{i}+d_{2}\overset{n}{\underset{j=1}{\sum }}A_{ij}\left(y_{j}-qy_{i}\right),\\ \frac{dz_{i}}{dt}=rsx_{i}-rz_{i}, \end{array}$$ (3)

where *A*_*ij*_ is the symmetric adjacent matrix to show the interaction of nodes on network. *x*_*j*_ − *qx*_*i*_, *y*_*j*_ − *qy*_*i*_ is the linear part of *f* (*x*_*j*_, *x*_*i*_), *g* (*y*_*j*_, *y*_*i*_), respectively. *q* can be treated as the difference between the collected current and the outgoing current. $L_{ij}=A_{ij}-q\overset{n}{\underset{j=1}{\sum }}A_{ij}$ and $\overset{n}{\underset{j=1}{\sum }}L_{ij}v_{j}^{k}=\Lambda_{k}v_{i}^{k}$ , $\Lambda_{k},v^{k}={\left(v_{1}^{k},\ldots ,v_{n}^{k}\right)}^{T}$ are the *k*th eigenvalue of *L* and the corresponding eigenvector, respectively Zheng and Shen (2020). If the coefficient of *x*_*j*_ does not equal to 1, it simplifies to 1 by extracting the common factors.

A general solution of system (3) is

$$\begin{array}{c} x_{i}=\overset{n}{\underset{k=1}{\sum }}c_{k}^{1}e^{\lambda_{k}t}v_{i}^{k},\\ y_{i}=\overset{n}{\underset{k=1}{\sum }}c_{k}^{2}e^{\lambda_{k}t}v_{i}^{k},\\ z_{i}=\overset{n}{\underset{k=1}{\sum }}c_{k}^{3}e^{\lambda_{k}t}v_{i}^{k}. \end{array}$$ (4)

Substituting system (4) into system (3), one has

$$\lambda_{k}\left(\begin{array}{c} c_{k}^{1}\\ c_{k}^{2}\\ c_{k}^{3} \end{array}\right)=J\left(\begin{array}{c} c_{k}^{1}\\ c_{k}^{2}\\ c_{k}^{3} \end{array}\right),$$

where

$$J=\left[\begin{array}{ccc} -3\;a{x_{0}}^{2}+d_{1}\Lambda +2\;bx_{0} & 1 & -e^{-\lambda_{k}\;\tau }\\ -2\;dx_{0} & d_{2}\Lambda -1 & 0\\ rs & 0 & -r \end{array}\right]$$

Then the characteristic equation is

$$\begin{array}{c} |\lambda_{k}E-J|=\lambda^{3}+a_{1}\lambda^{2}+\left(a_{2}+a_{3}e^{-\lambda \;\tau }\right)\lambda +a_{4}+a_{5}e^{-\lambda \;\tau }=0, \end{array}$$ (5)

where

$$\begin{array}{c} a_{1}=3\;a{x_{0}}^{2}-2\;bx_{0}-\Lambda_{k}d_{1}-\Lambda_{k}d_{2}+r+1,\\ a_{2}=-3\;a{x_{0}}^{2}\Lambda_{k}d_{2}+3\;ar{x_{0}}^{2}+2\;bx_{0}\Lambda_{k}d_{2}+{\Lambda_{k}}^{2}d_{1}d_{2}+3\;a{x_{0}}^{2}-2\;brx_{0}-r\Lambda_{k}d_{1}-r\Lambda_{k}d_{2}-2\;bx_{0}+2\;dx_{0}-\Lambda_{k}d_{1}+r,\\ a_{3}=rs,\\ a_{4}=-3\;ar{x_{0}}^{2}\Lambda_{k}d_{2}+2\;brx_{0}\Lambda_{k}d_{2}+r{\Lambda_{k}}^{2}d_{1}d_{2}+3\;ar{x_{0}}^{2}-2\;brx_{0}+2\;dx_{0}r-r\Lambda_{k}d_{1},\\ a_{5}=-rs\Lambda_{k}d_{2}+rs. \end{array}$$

When *τ* = 0, the characteristic equation is,

$$\begin{array}{c} p\left(\lambda \right)=\lambda^{3}+a_{1}\lambda^{2}+\left(a_{2}+a_{3}\right)\lambda +a_{4}+a_{5}=0. \end{array}$$ (6)

According to Hurwitz criterion, the sufficient and necessary conditions for stable system (1) are,

$$\begin{array}{c} a_{1}>0,a_{2}+a_{3}>0,a_{4}+a_{5}>0,a_{1}\left(a_{2}+a_{3}\right)-\left(a_{4}+a_{5}\right)>0. \end{array}$$ (7)

The converse-negative proposition is the condition of Turing instability, namely, Turing instability induced by network when one of *H*_*i*_(*i* = 1, 2, 3) holds.

$$\begin{array}{c} H_{1}:a_{2}+a_{3}<0,\\ H_{2}:a_{4}+a_{5}<0,\\ H_{3}:a_{1}\left(a_{2}+a_{3}\right)-\left(a_{4}+a_{5}\right)<0. \end{array}$$

More precisely, for *H*_1_, Turing instability occurs when

$$\begin{array}{c} p_{1}\left(\Lambda_{k}\right)=a_{2}+a_{3}=b_{1}\Lambda_{k}^{2}+b_{2}\Lambda_{k}+b_{3}<0 \end{array}$$

where

$$\begin{array}{c} b_{1}=d_{1}d_{2},b_{2}=-3\;ad_{2}{x_{0}}^{2}+2\;bd_{2}x_{0}-rd_{1}-rd_{2}-d_{1},\\ b_{3}=3\;ar{x_{0}}^{2}+3\;a{x_{0}}^{2}-2\;brx_{0}-2\;bx_{0}+2\;dx_{0}+rs+r. \end{array}$$

According to the properties of quadratic equations of one variable, the minimum value of *p*_1_ (Λ_*k*_) is $p_{1}\left(-\frac{b_{2}}{2b_{1}}\right)$ ; if $-\frac{b_{2}}{2b_{1}}>0$ and *p*_1_ (0) < 0, Turing instability occurs; if $-\frac{b_{2}}{2b_{1}}<0$ and $p_{1}\left(-\frac{b_{2}}{2b_{1}}\right)<0$ , Turing instability occurs and the critical value is $\Lambda_{k}=-\frac{b_{2}}{2b_{1}}$ .

For *H*_2_, Turing instability occurs when

$$\begin{array}{c} p_{2}\left(\Lambda_{k}\right)=a_{4}+a_{5}=c_{1}\Lambda_{k}^{2}+c_{2}\Lambda_{k}+c_{3}<0 \end{array}$$

where, the analysis process refers to *p*_1_ (Λ_*k*_), and

$$\begin{array}{c} c_{1}=rd_{1}d_{2},c_{2}=-3\;ard_{2}{x_{0}}^{2}+2\;brd_{2}x_{0}-rsd_{2}-rd_{1},\\ c_{3}=3\;ar{x_{0}}^{2}-2\;brx_{0}+2\;dx_{0}r+rs. \end{array}$$

For *H*_3_, Turing instability occurs when

$$\begin{array}{c} p_{3}\left(\Lambda_{k}\right)=-\left[a_{1}\left(a_{2}+a_{3}\right)-a_{4}+a_{5}\right]=q_{1}\Lambda_{k}^{3}+q_{2}\Lambda_{k}^{2}+q_{3}\Lambda_{k}+q_{4}>0 \end{array}$$

where

$$\begin{array}{cc} q_{1} & ={d_{1}}^{2}d_{2}+d_{1}{d_{2}}^{2},\\ q_{2} & =-6\;ad_{1}d_{2}{x_{0}}^{2}-3\;a{d_{2}}^{2}{x_{0}}^{2}+4\;bd_{1}d_{2}x_{0}+2\;b{d_{2}}^{2}x_{0}-r{d_{1}}^{2}-2\;rd_{1}d_{2}-r{d_{2}}^{2}-{d_{1}}^{2}-2\;d_{1}d_{2},\\ q_{3} & =9\;a^{2}d_{2}{x_{0}}^{4}-12\;abd_{2}{x_{0}}^{3}+6\;ard_{1}{x_{0}}^{2}+6\;ard_{2}{x_{0}}^{2}+4\;b^{2}d_{2}{x_{0}}^{2}+6\;ad_{1}{x_{0}}^{2}\\ & \;+6\;ad_{2}{x_{0}}^{2}-4\;brd_{1}x_{0}-4\;brd_{2}x_{0}-4\;bd_{1}x_{0}-4\;bd_{2}x_{0}+2\;dd_{1}x_{0}\\ & \;+2\;dd_{2}x_{0}+rsd_{1}+r^{2}d_{1}+r^{2}d_{2}+2\;rd_{1}+2\;rd_{2}+d_{1},\\ q_{4} & =-9\;a^{2}r{x_{0}}^{4}-9\;a^{2}{x_{0}}^{4}+12\;abr{x_{0}}^{3}+12\;ab{x_{0}}^{3}-6\;ad{x_{0}}^{3}-3\;ars{x_{0}}^{2}\\ & \;-3\;ar^{2}{x_{0}}^{2}-4\;b^{2}r{x_{0}}^{2}-6\;ar{x_{0}}^{2}-4\;b^{2}{x_{0}}^{2}+4\;bd{x_{0}}^{2}+2\;brsx_{0}+2\;br^{2}x_{0}\\ & \;-3\;a{x_{0}}^{2}+4\;brx_{0}-sr^{2}+2\;bx_{0}-2\;dx_{0}-r^{2}-r. \end{array}$$

Suppose *r*_1_, *r*_2_ (*r*_1_ ≤ *r*_2_) is the roots of $p_{3}^{\prime }\left(\Lambda_{k}\right)$ ,

$$\begin{array}{c} p_{3}^{\prime }\left(\Lambda_{k}\right)=3q_{1}\Lambda_{k}^{2}+2q_{2}\Lambda_{k}+q_{3} \end{array}$$

where *p*_3_ (*r*_1_), *p*_3_ (*r*_2_) are local maximum and minimum respectively. According to the properties of cubic equations of one variable, Turing instability occurs when *r*_1_ < 0 and *p*_3_ (*r*_1_) > 0; Turing instability occurs when *r*_1_ > 0 and *p*_3_ (0) > 0.

Finally, we consider the stability of system (1), namely,

$$\begin{array}{c} p_{4}\left(\lambda \right)=\lambda^{3}+a_{1}\lambda^{2}+\left(a_{2}+a_{3}e^{-\lambda \;\tau }\right)\lambda +a_{4}+a_{5}e^{-\lambda \;\tau }=0. \end{array}$$ (8)

Suppose that the pure imaginary root *λ* = *j ω* (*j* represents the imaginary unit) exists and we substitute it into the above characteristic equation, we have

$$-a_{1}\omega^{2}+\sin\left(\omega \;\tau \right)\omega \;a_{3}+a_{4}+\cos\left(\omega \;\tau \right)a_{5}+\left(-\omega^{3}+\left(a_{2}+\cos\left(\omega \;\tau \right)a_{3}\right)\omega -\sin\left(\omega \;\tau \right)a_{5}\right)j=0.$$

Separating the real and imaginary parts

$$\begin{array}{c} -a_{1}\omega^{2}+\sin\left(\omega \;\tau \right)\omega \;a_{3}+a_{4}+\cos\left(\omega \;\tau \right)a_{5}=0,\\ -\omega^{3}+\left(a_{2}+\cos\left(\omega \;\tau \right)a_{3}\right)\omega -\sin\left(\omega \;\tau \right)a_{5}=0. \end{array}$$

Solving cos (*ωτ*), sin (*ωτ*) to get

$$\begin{array}{c} \cos\left(\omega \tau \right)=\frac{\omega^{4}a_{3}+\omega^{2}a_{1}a_{5}-\omega^{2}a_{2}a_{3}-a_{4}a_{5}}{\omega^{2}{a_{3}}^{2}+{a_{5}}^{2}},\\ \sin\left(\omega \tau \right)=\frac{\omega \;\left(\omega^{2}a_{1}a_{3}-\omega^{2}a_{5}+a_{2}a_{5}-a_{3}a_{4}\right)}{\omega^{2}{a_{3}}^{2}+{a_{5}}^{2}}. \end{array}$$

Due to cos (*ωτ*)^2^ + sin (*ωτ*)^2^ = 1, one has

$$\begin{array}{c} s_{1}x^{4}+s_{2}x^{3}+s_{3}x^{2}+s_{4}x+s_{5}=0, \end{array}$$ (9)

where

$$\begin{array}{c} x=\omega^{2},s_{1}={a_{3}}^{2},\\ s_{2}={a_{1}}^{2}{a_{3}}^{2}-2\;a_{2}{a_{3}}^{2}+{a_{5}}^{2},\\ s_{3}={a_{1}}^{2}{a_{5}}^{2}-2\;a_{1}{a_{3}}^{2}a_{4}+{a_{2}}^{2}{a_{3}}^{2}-{a_{3}}^{4}-2\;a_{2}{a_{5}}^{2},\\ s_{4}=-2\;a_{1}a_{4}{a_{5}}^{2}+{a_{2}}^{2}{a_{5}}^{2}+{a_{3}}^{2}{a_{4}}^{2}-2\;{a_{5}}^{2}{a_{3}}^{2},\\ s_{5}={a_{4}}^{2}{a_{5}}^{2}-{a_{5}}^{4}. \end{array}$$

If a positive root exists *x*_*i*_ (*i* = 1, 2, 3, 4) at least in system (9), one has

$$\begin{array}{c} \tau_{i}=\frac{1}{\omega_{i}}\arccos\left(\frac{\omega^{4}a_{3}+\omega^{2}a_{1}a_{5}-\omega^{2}a_{2}a_{3}-a_{4}a_{5}}{\omega^{2}{a_{3}}^{2}+{a_{5}}^{2}}\right)+\frac{2\pi }{\omega_{i}}, \end{array}$$

where $\omega_{i}=\sqrt{x_{i}}$ is the solution of system (9) and the critical value *τ*_0_ = min{*τ*_*i*_} when *d*_1_ = *d*_2_ = 0, and *τ*_*c*_ = min{*τ*_*i*_} when *d*_1_ ≠ 0 or *d*_2_ ≠ 0. Also, the corresponding *ω* of *τ*_0_, *τ*_*c*_ is *ω*_0_, *ω*_*c*_.

The transversality condition

$$\begin{array}{c} \frac{d\lambda }{d\tau }=\frac{t_{3}cos\left(\omega \tau \right)+t_{4}sin\left(\omega \tau \right)+t_{5}}{t_{1}^{2}+t_{2}^{2}}\neq 0, \end{array}$$

where

$$\begin{array}{c} t_{1}=-\sin\left(\omega \;\tau \right)\omega \;\tau \;a_{3}+\cos\left(\omega \;\tau \right)\tau \;a_{5}+3\;\omega^{2},\\ t_{2}=\cos\left(\omega \;\tau \right)\omega \;\tau \;a_{3}+\sin\left(\omega \;\tau \right)\tau \;a_{5}-2\;a_{1}\omega ,\\ t_{3}=-3\;a_{3}\omega^{4}-2\;\omega^{2}a_{1}a_{5}-3\;a_{3}\omega^{2}-\tau \;a_{2}a_{5},\\ t_{4}=2\;\omega^{3}a_{1}a_{3}-3\;\omega^{3}a_{5}+\omega \;\tau \;a_{2}a_{3}+2\;\omega \;a_{1}a_{3},\\ t_{5}=-3\;a_{2}\omega^{2}-a_{3}\tau \;a_{5}. \end{array}$$

If

$$\begin{array}{c} \frac{d\lambda }{d\tau }{|}_{\tau =\tau_{c},\omega =\omega_{c}}>0, \end{array}$$

Turing instability occurs when *τ*_0_ > *τ* > *τ*_*c*_. *τ*_0_ > *τ* make system (1) without network stable, and network induce Turing instability, namely, *τ*_0_ > *τ* > *τ*_*c*_.

Through the above analysis, we can draw the following sufficient conditions for Turing instability.

Theorem 1 Turing instability occurs in the network-organized system when a *H*_*i*_ (*i* = 1, 2, 3) holds; Turing instability occurs in the network-organized system with delay when *τ* ∈ (*τ*_0_, *τ*_*c*_).

Proof 1 The proof process can refer to the above analysis.

## Results and Discussion

In this section, these parameters *a* = 1, *b* = 3, *c* = 1, *d* = 5, *r* = 0.01, s = 4, *I* = 1, *x*_*r*_ = −1.6 Hindmarsh and Rose (1984) and *d*_1_ = 0.1, *d*_2_ = 0.3 are set. The adjacent matrix *A* is generated by random network Zheng and Shen (2020); Erdos and Renyi (1959) and the link probability *p* and nodes *n* = 100 is the initial value. For node *i* and node *j*, if the random number <p , *A*_*ij*_ = *A*_*ji*_ = 1, or else *A*_*ij*_ = *A*_*ji*_ = 0. Suppose the special form *f* (*x*_*j*_, *x*_*i*_) = *x*_*j*_ − *qx*_*i*_, *g* (*y*_*j*_, *y*_*i*_) = *y*_*j*_ − *qy*_*i*_ in system (1). Also, *x* represent 100 neuron nodes in the pattern formation.

**FIGURE 1:**
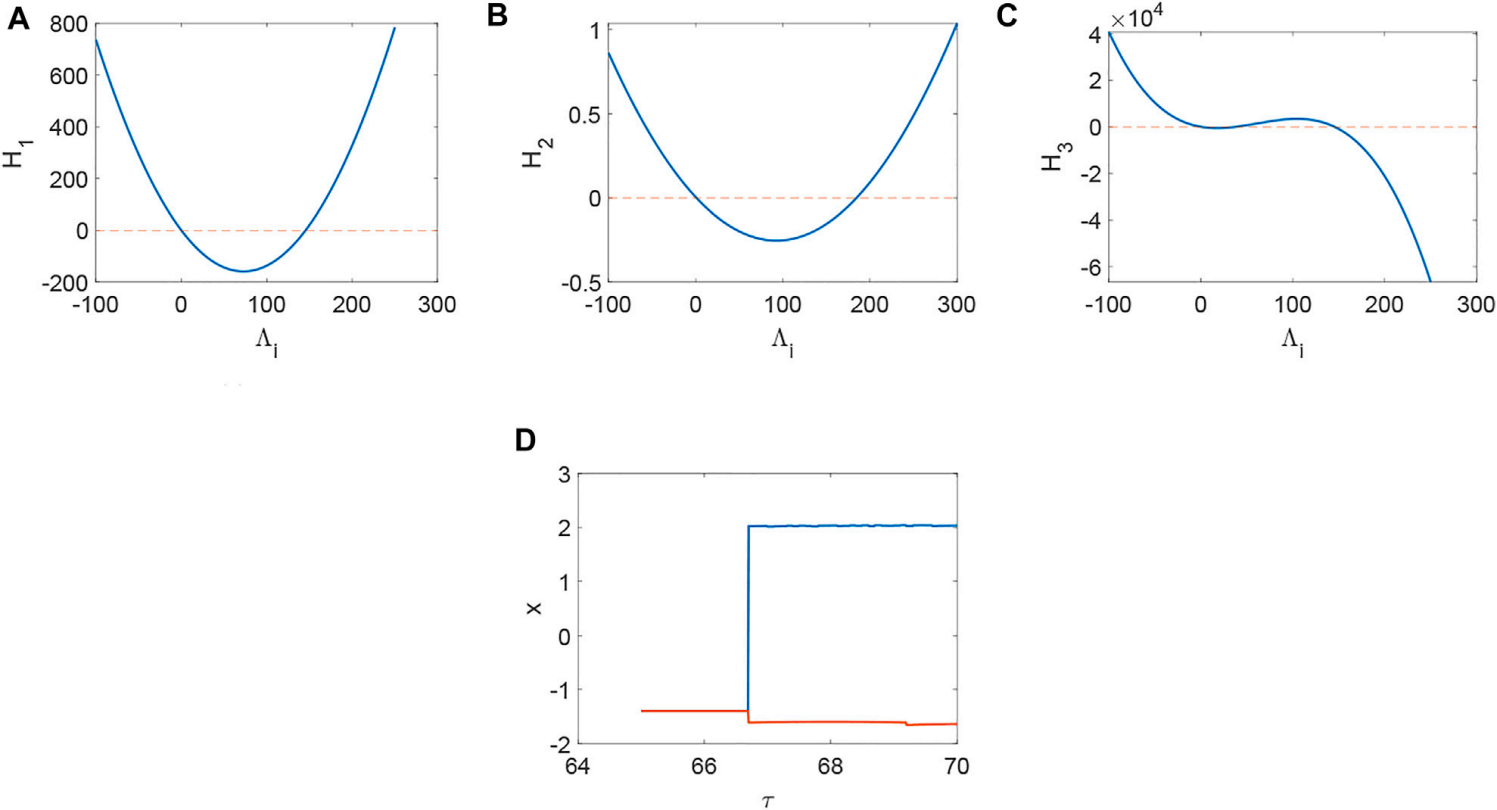
The distribution of *H*_*i*_(*i* = 1, 2, 3) when *a* = 1, *b* = 3, *c* = 1, *d* = 5, *r* = 0.01, s = 4, *I* = 1, *x*_*r*_ = −1.6, *d*_1_ = 0.1, *d*_2_ = 0.3, *τ* = 0. **(A)** Turing instability occurs when *H*_1_ < 0, or else stable. **(B)** Turing instability occurs when *H*_2_ < 0, or else stable. **(C)** Turing instability occurs when *H*_3_ < 0, or else stable. **(D)** The bifurcation about *τ* when *d*_1_ = *d*_2_ = 0.

**FIGURE 2:**
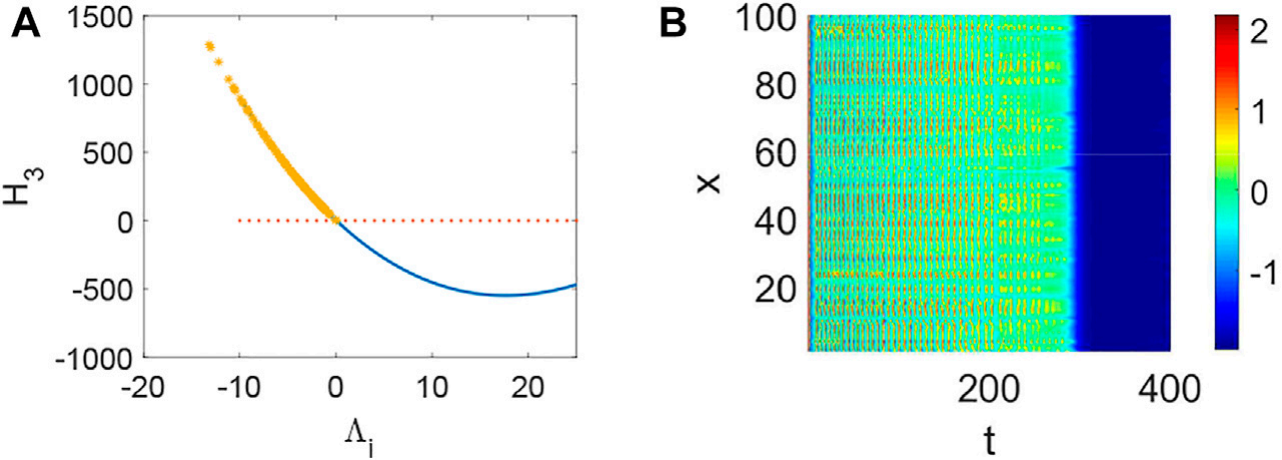
The stability of system (1) when *p* = 0.05, *τ* = 0. **(A)** The distribution of eigenvalues Λ_*i*_ (labeled by *) when *q* = 0. **(B)** The corresponding pattern formation is stable in system (1) when no eigenvalue Λ_*i*_ make *H*_3_ < 0 hold.

**FIGURE 3:**
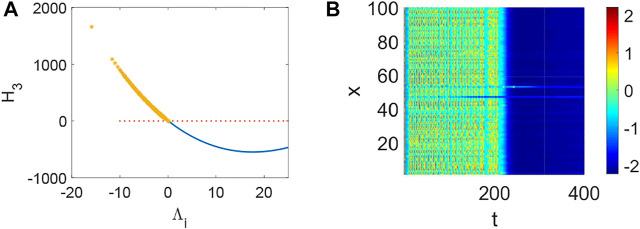
The stability of system (1) when *p* = 0.05, *q* = 0.96, *τ* = 0. **(A)** The distribution of eigenvalues Λ_*i*_ (labeled by *). **(B)** Turing instability occurs in system (1) when some eigenvalue Λ_*i*_ make *H*_3_ < 0 hold.

**FIGURE 4:**
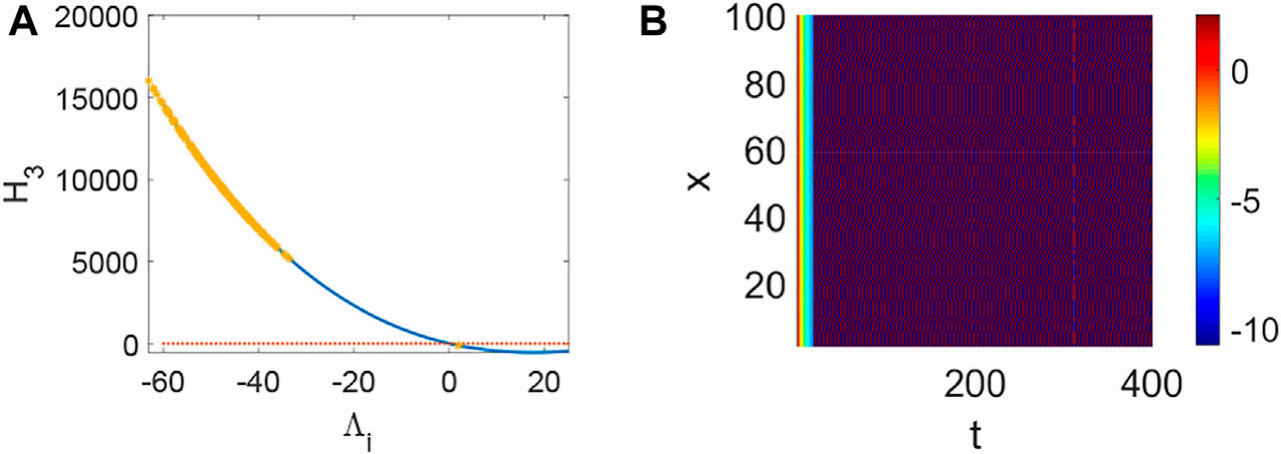
The stability of system (1) when *p* = 0.5, *q* = 0.96, *τ* = 0. **(A)** The distribution of eigenvalues Λ_*i*_ (labeled by *). **(B)** Turing instability occurs in system (1) when a eigenvalue Λ_*i*_ make *H*_3_ < 0 hold.

**FIGURE 5:**
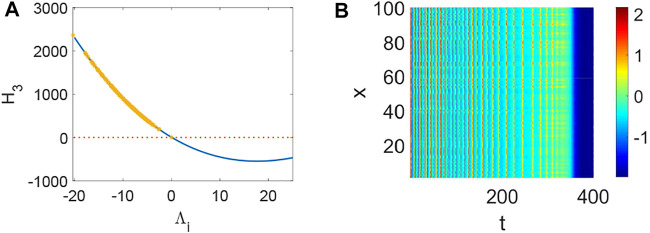
The stability of system (1) when *p* = 0.1, *q* = 0, *τ* = 0. **(A)** The distribution of eigenvalues Λ_*i*_ (labeled by *). **(B)** The corresponding pattern formation is stable in system (1) when no eigenvalue Λ_*i*_ make *H*_3_ < 0 hold.

**FIGURE 6:**
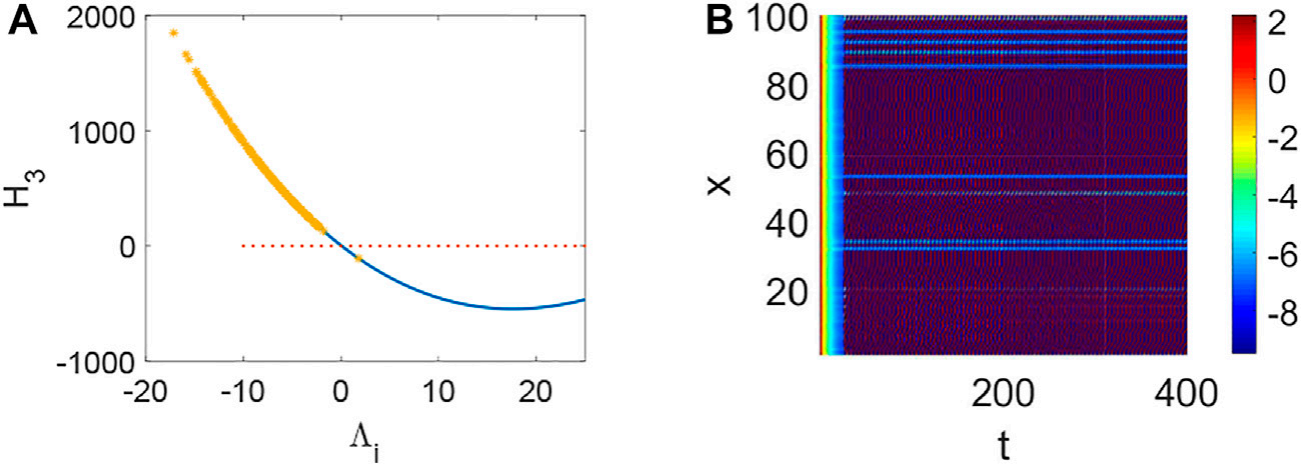
The stability of system (1) when *p* = 0.1, *q* = 0.82, *τ* = 0. **(A)** The distribution of eigenvalues Λ_*i*_ (labeled by *). **(B)** Turing instability occurs in system (1) when some eigenvalue Λ_*i*_ make *H*_3_ < 0 hold.

**FIGURE 7:**
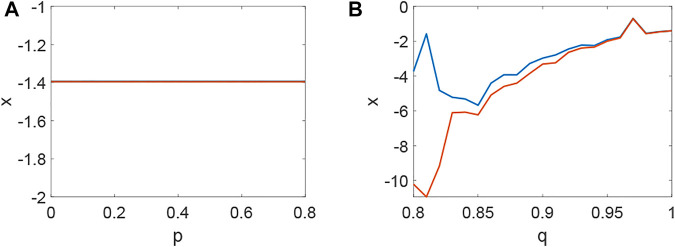
The bifurcation about *p*, *q* when *τ* = 0. **(A)** The inexistence bifurcation about *p* when *q* = 0. **(B)** The bifurcation about *q* when *p* = 0.1.

From Figure 1, system (1) is stable when *d*_1_ = 0, *d*_2_ = 0, *τ* = 0, which is the precondition of Turing instability induced by network and delay. Namely, *H*_*i*_ > 0 when Λ_*i*_ = 0 (Figure 1A,B,C) and the current values below the activity threshold of the single neuron. Also, Λ_*i*_ can not lead to Turing instability when Λ_*i*_ < 0, which means it is difficult to keep the network in activity when the collected current is the same as the outgoing current. Let’s take *H*_3_ (*H*_1_ and *H*_2_ are relatively simple, and *H*_3_ is more representative.) as an example to illustrate the stability of system (1) (Figure 2–6). Although system (1) without delay is periodic behavior (Figure 2B) under the initial stimulus, it ultimately tends to the rest state (Figure 2A) when no stimulus is added. Short-term memory attributes to a fixed point attractor Wang (2001), which could persistent neuronal activity when the remembered stimulus is removed Goldman (2009). The above results bring into correspondence with our results (Figure 2). When the collected current is larger than the outgoing current*q* = 0.96, *H*_3_ < 0 holds (Figure 3A) and Turing instability occurs (Figure 3B). In general, most of the neurons will be in the rest state. Meanwhile, a few persistent neuronal activities because of the constant external stimuli (Figure 3B). When the link probability *p* increases and the difference *q* becomes larger, *H*_3_ < 0 holds (Figure 4A) and the corresponding pattern formation (Figure 4B) shows the periodic neural activity. Namely, constant external stimulation from other neurons is necessary to keep the neural activity. For the short-term memory, noise could induce the switch of different memories Zheng et al. (2020), and the constant external stimulation from other neurons can also leads to the switch of different memories. Only the link probability could not causes instability (Figure 5A), but it could keep the neural activity longer (Figure 5B). When the difference between the collected current and the outgoing current exceeds the threshold value of the neural activity (Figure 6A), Turing instability occurs (Figure 6B). Namely, the short-term memory generation requires enough stimulation, which is in line with the actual situation. Also, these above results can be verified by the bifurcation (Figure 7):system (1) is always stable when *q* = 0 (Figure 7A) and *q* could induce Turing instability (Figure 7B).

**FIGURE 8:**
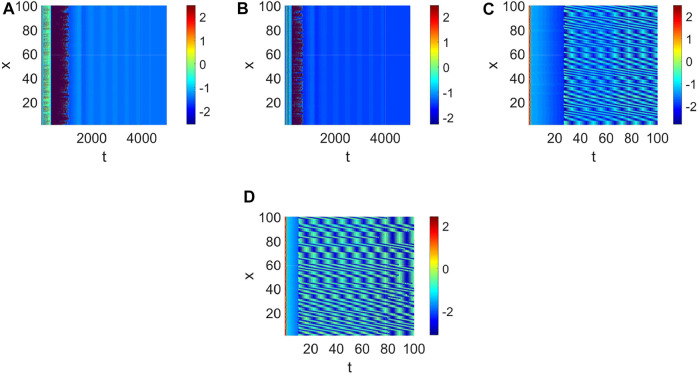
The pattern formation of system (1) when *τ* = 65. **(A)** The pattern formation when *p* = 0.05, *q* = 0. **(B)** The pattern formation when *p* = 0.1, *q* = 0. **(C)** The pattern formation when *p* = 0.1, *q* = 0.96. **(D)** The pattern formation when *p* = 0.15, *q* = 0.96.

**FIGURE 9:**
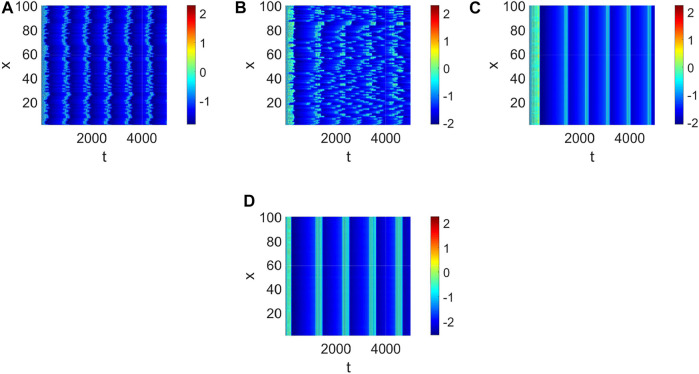
The pattern formation of system (1) when *τ* = 68. **(A)** The pattern formation when *p* = 0, *q* = 0. **(B)** The pattern formation when *p* = 0.01, *q* = 0. **(C)** The pattern formation when *p* = 0.1, *q* = 0. **(D)** The pattern formation when *p* = 0.1, *q* = 0.96.

**FIGURE 10:**
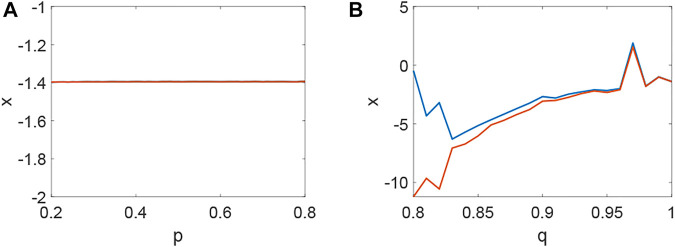
The bifurcation about *p*, *q* when *τ* = 65. **(A)** The inexistence bifurcation about *p* when *q* = 0. **(B)** The bifurcation about *q* when *p* = 0.1.

Delay plays a vital role in the rise or fall of neural activity. From Figure 8, the dynamic behavior (Figure 8A) is different from system (1) without delay (Figure 2), but the link probability nearly has no effect on the stability of system (1) (Figure 8B), because the collected current always equal to the outgoing current when *q* = 0. When *q* ≠ 0, the periodic behavior of the neuron may occur (Figure 8C,D). Meanwhile, the periodic behavior of the neuron under different *p* (Figure 8C,D). It is found that the link probability *p* could make system (1) network synchronization and the synchronicity becomes stronger with the increase of *p*, but the neuronal activity is low (Figure 9A,B,C, Figure 10A). Of course, *q* could increases neuronal activity (Figure 9D), which can also be represented in the bifurcation (Figure 10B). The phenomenon of spatiotemporal patterns Lepek and Fronczak (2018); Umesh and Ambika (2021); Shi et al. (2022); Santos et al. (2017) was often considered in the network-organized system when *q* = 1. The difference *q* < 1 between the collected current and the outgoing current plays a vital role in the neuronal activity through the above analysis, which may further show the firing mechanism in a general networked HR model with delay. Meanwhile, short-term memory results from external stimuli Zheng et al. (2020), but not all stimuli result in short-term memory. The difference *q* < 1 may theoretically explain why some stimuli can’t lead to short-term memory.

## Conclusion

In this paper, spatiotemporal patterns are investigated to illustrate the collected behavior of neurons and the generation mechanism of short-term memory. We obtain the algebraic expressions for Turing instability to occur in any HR network setup. Then, we derive the critical value of Hopf bifurcation to present a profound impact of both network and delay on the Turing instability. Also, we find that the collected current and outgoing current play a vital role in neuronal activity, especially in the generation mechanism of the short-term memory. Meanwhile, the collected behavior may fire when the input to the neuron is below a certain threshold, and the output reaches a stationary regime. Finally, we try to explain the generation mechanism of the short-term memory through the theoretical results and numerical simulation.

## Data Availability

The original contributions presented in the study are included in the article, further inquiries can be directed to the corresponding author (Some related code can be found: https://github.com/zhengqianqian35).
